# Improving data use for decision making by neglected tropical disease program teams: eight use cases

**DOI:** 10.12688/gatesopenres.13407.1

**Published:** 2021-11-09

**Authors:** Liz Grubin, Lakshmi Balachandran, Sarah Bartlett, Nana-Kwadwo Biritwum, Simon Brooker, Fiona Fleming, Karsor Kollie, Sultani Matendechero, Birhan Mengistu, TJ Muehleman, Upendo Mwingira, Brooke Partridge, Alex Pavluck, Maria Rebollo Polo, Modeste Tezembong, Derek Treatman, Rosalyn Yeary, Katie Zoerhoff, Honorat Zoure

**Affiliations:** 1 Vital Wave, Palo Atlo, CA, USA; 2 Clinton Health Access Initiative, Boston, MA, USA; 3 Sightsavers, Haywards Heath, UK; 4 Bill & Melinda Gates Foundation, Seattle, WA, USA; 5 SCI Foundation, London, UK; 6 Non-Communicable and Neglected Tropical Disease Program, Ministry of Health and Social Welfare, Monrovia, Liberia; 7 Division of Vector Borne and Neglected Tropical Diseases, Kenya Ministry of Health, Nairobi, Kenya; 8 Neglected Tropical Diseases, Federal Ministry of Health of Ethiopia, Addis Ababa, Ethiopia; 9 Standard Code, Atlanta, USA; 10 Tanzanian Neglected Tropical Diseases Control Programme, Ministry of Health, Dar es Salaam, Tanzania; 11 Expanded Special Project on the Elimination of Neglected Tropical Diseases, WHO-AFRO, Brazzaville, Congo; 12 RTI International, Research Triangle Park, NC, USA

**Keywords:** Neglected tropical diseases; data systems; decision-making; use cases; data solutions

## Abstract

**Background: **The achievement of neglected tropical diseases (NTDs) program goals depends on numerous factors, including the ability of national programs to use high-quality, timely data to inform their decision-making and program delivery. This paper presents a use case analysis of the routine data used by national NTD programs targeting lymphatic filariasis, onchocerciasis, schistosomiasis, soil-transmitted helminthiasis, and trachoma.

**Methods:** The use cases were developed through a combination of secondary and primary research focused on both global trends and deep dives into Burkina Faso, Ethiopia, and Tanzania. Results were refined through a stakeholder convening and the final eight use cases were determined through iteration and prioritization with stakeholders.

**Results:** Eight use cases were developed: improve treatment register data quality, strengthen supervision of drug distributors during mass drug administration (MDA), generate accurate community-level population data for MDAs, create and manage an accurate inventory of drugs, meet district coverage targets during MDA campaigns, feedback and performance to sub-district teams, feedback on performance to sub-national teams, and national-level program use of data for evaluation and decision making. Each use case identifies key actors and their data-related needs and critical challenges, defines the current and desired state, and articulates the profile of a solution (digital and non-digital) needed to complete the use case.

**Conclusion:** The systematic strengthening of data use for decision-making in NTD programs is key for reaching the 2030 Roadmap goals. Integrated together, the presented use cases, when translated into action using appropriate and innovative solutions, can help to ensure that accurate and timely data are present at every step of a program and empower countries to use these data to make program decisions.

## Introduction

Since 2010, the global community has made strong progress in the prevention, control, and elimination of neglected tropical diseases (NTDs). The number of people requiring NTD interventions has declined by 500 million, and more than 40 countries have eliminated at least one NTD ( The Lancet Global Health, 2020; WHO, 2020a). The year 2020 saw the launch of a new WHO roadmap for 2021–2030, which outlines targets and milestones in the fight against 20 NTDs. Achievement of these targets depends on numerous factors, including the ability of national programs to use high-quality, timely data to inform their decision-making and program delivery. In particular, programs need to (i) determine where to target mass drug administration (MDA) and other interventions, (ii) undertake planning and resource-allocation, (iii) detect and address poorly performing areas, (iv) determine where there is a need to change intervention strategy or whether interventions can be stopped, and (v) how to implement post-MDA surveillance, among others. If programs cannot access relevant data or do not trust the quality of available data, they will not use them; and if they use low-quality data, they may make incorrect decisions that will ultimately hamper progress towards programmatic targets. 

There are several reasons as to why programs do not use the best available data to make decisions including: NTD data sources and health information systems are often fragmented, which makes it difficult for data users to access relevant data; data collection and data management may be prone to technical or human error ( de Souza *et al*., 2016); and technical and institutional barriers to sharing of data may exist. Improved data sources, tools, and systems can help address some of these issues, but alone they will not be sufficient to ensure data are used effectively; there is a need to strengthen the capacity of individuals working at all levels of the health system and to couple that with a supportive enabling environment.

The Global Strategy on Digital Health, 2020–2024 developed by the World Health Organization (WHO) defines seven components of such enabling environments ( WHO, 2020b), based upon the WHO’s health system strengthening building blocks, with an emphasis on standards and interoperability, workforce, and leadership and governance ( WHO, 2010). In the context of NTDs, the WHO Roadmap, 2021–2030 emphasizes the need for integrated, digital data systems that support decision-making at national and local levels and which are interoperable with national health information systems ( WHO, 2020a).

To understand opportunities to improve data use within a given context, it is important to identify the main decisions faced by NTD programs and the specific situations in which data are used – or not used – by different individuals. In particular, it is instructive to understand the challenges faced by individuals in using data in their decision-making and the broader implications of these challenges for NTD programs, as this can help identify gaps and appropriate solutions. Here, a useful framework is use case analysis which is commonly used in diagnostic, pharmaceutical and other industries to define the intended use of a technology or data type, identify key actors and their needs with regards to a solution, and determine the key features and capabilities that solutions will need to fully address the use case ( Hunt, 1999).

This paper presents eight priority data use cases for NTD programs targeting lymphatic filariasis, onchocerciasis, schistosomiasis, soil-transmitted helminths, and trachoma. The use cases are designed to provide NTD stakeholders with information to improve the accuracy, completeness and timeliness of NTD data and to understand where new data solutions (digital and non-digital) could improve data-driven decision-making. 

## Methods

Between 2017 and 2018, the Bill & Melinda Gates Foundation (the foundation) partnered with Vital Wave to engage national NTD programs and the global NTD community to define a series of data use cases in MDA campaigns. Development of the use cases arose from four phases of work focused on the data use and data needs of NTD program actors involved in MDA campaigns.

The first phase of research informed the broader context for NTD program activities and priorities, identifying common data needs and challenges. This phase included literature reviews and semi-structured interviews with 26 international stakeholders including NTD experts, implementing partners, the World Health Organization, donors, and technology experts. The literature review was based on a structured search of the online database PubMed using key MeSH terms related to NTDs and data systems and data use. Grey literature was also sought through abstract searches and internet searches. The international stakeholders were identified at the COR-NTD conference in 2017 and interviews were scheduled either at the meeting or soon afterwards. An interview guide was used and included prompts on a number of key areas for discussion, including the main objectives and activities in completing MDAs, the different types of data sources that each stakeholder type relied on, current challenges or advantages of those data sources, and the different country-level actors engaged in NTD program activities.

The second phase assessed country-level use of NTD data and focused on further detailing and validating the process and data flow for MDA campaigns in Burkina Faso, Ethiopia, and Tanzania. In-country research conducted in 2017 and early 2018 focused on identifying each data-related step taken by relevant actors responsible for MDA implementation. Research was conducted by Vital Wave in Burkina Faso and Tanzania, while RTI International undertook similar work in Ethiopia. In-person interviews and focus group discussions were conducted with actors at all levels of the health system, including national NTD program managers and data managers, pharmacists at the regional and district level, health workers at the district and facility level, and drug distributors and teachers responsible for administering MDAs in communities and schools. The assessments characterized the main NTD data flows and their bottlenecks as well as root causes and implications of identified bottlenecks. The assessment also identified potential solutions.

Findings from both the stakeholder interviews and in-country research formed the third phase, an expert strategic convening held in March 2018 and attended by interviewed stakeholders as well as other NTD program experts and technologists active in digital health. The convening was used to refine and validate the findings on critical challenges and priority focus areas, leading to the development of over 20 potential use cases.

Lastly, a prioritization process informed the selection of use cases, using a phased approach. Through a process of review, discussion, and iteration with the NTD team at the foundation, eight final use cases were selected. Once selected, the use cases were fully developed, with the finalization process including additional review from the foundation team and select stakeholders from the NTD global community at an in-person workshop during the Coalition for Operational Research on NTDs (COR-NTD) meeting in 2018.

The content of each data use case is comprised of four components: the objective, key actors, critical data challenges, and causal issues. The objective defines the overarching goal of the use case, essentially the “desired state” that appropriate solutions can help to realize. Key actors are the NTD program team members involved in the use case activities, and the data challenges are the data-related pain points they experience (the “current state”), which were mapped in a graphical data flow. Causal issues driving the critical challenges are identified, and the enabling environment component most essential to the use case is also defined.

## Results

**Figure 1.**
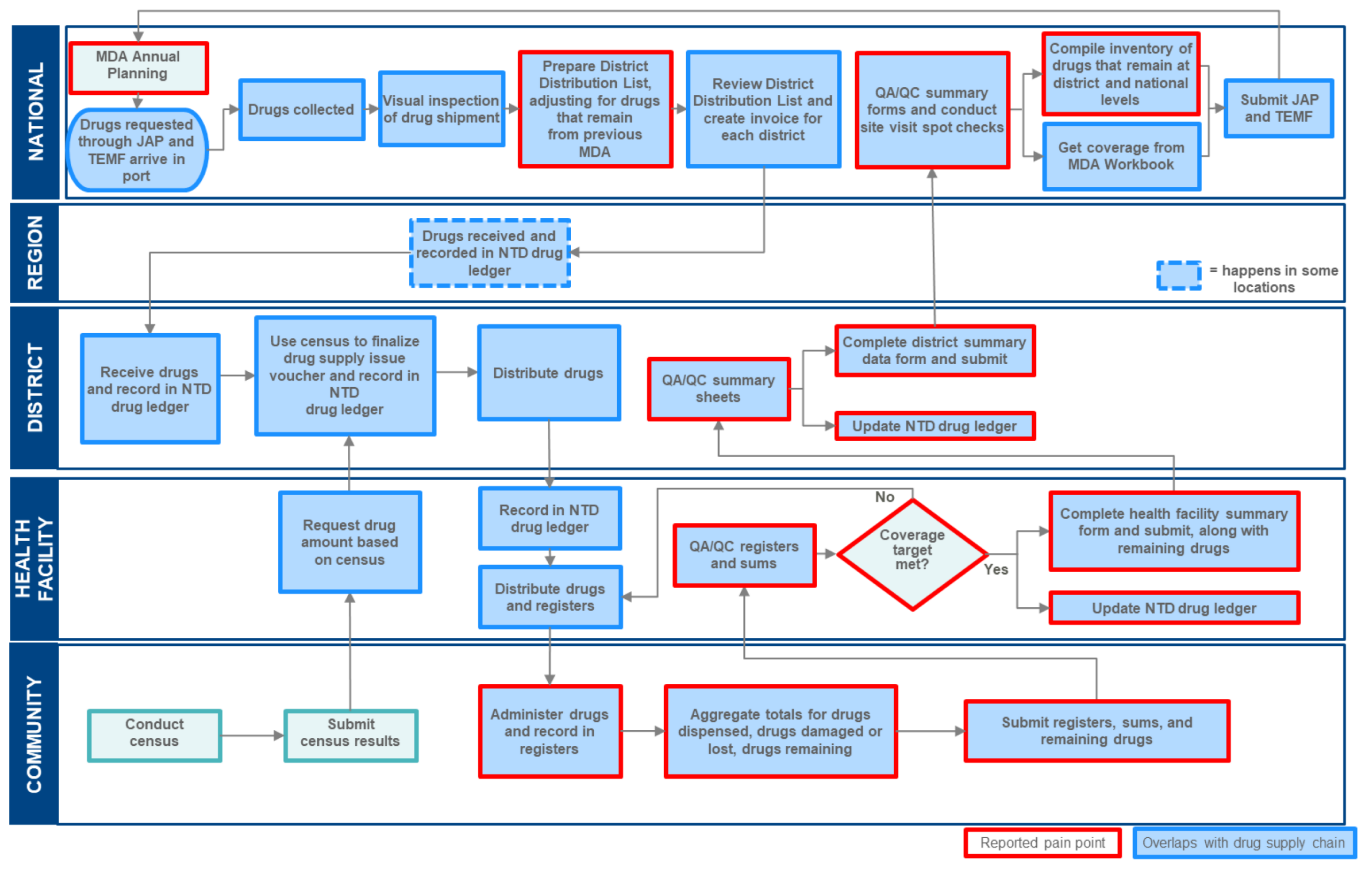
MDA campaign data flow.

Figure 1 presents the flow of data for a typical MDA campaign. The diagram depicts each data-related step and which actors, by health system level, are involved. Identified pain points are noted, representing steps where actors reported that their decision was compromised as a result of data-related challenges. The data flow provides a framework to identify different use cases.

The assessments identified common broad themes for data use by NTD programs. First, actors at every level of the health system depend on data to make good decisions but are inhibited by system bottlenecks, including lack of programmatic and technical system integration, an over-stretched and under-capacitated workforce, and limited financial resources. Second, data systems and tools used by NTD programs are limited in their ability to support using high-quality data for real-time decision making because of fragmented, non-interoperable systems that inhibit data sharing and lack visualization and analysis capabilities. Third, priority data sources for NTD programs are fragmented and have compromised data quality and access. Lastly, there are few incentives to use data in decision-making at different levels of NTD programs.

Four data sources were identified as having the greatest impact on programmatic decision-making: (i) community and treatment registers to record individuals who receive treatment during an MDA; (ii) official census data to estimate population requiring treatment and treatment coverage; (iii) drug ledgers to capture drug inventory before, during, and after MDA throughout the supply chain; and (iv) monitoring and evaluation (M&E) data to record routine MDA data, MDA monitoring data, and epidemiological survey data. While issues with inaccurate and delayed data for treatment registers originate at the community level, this poor data quality inhibits decision-making for NTD actors at all levels of the health system. A lack of acceptable denominator data (based on either census data, community registers, or both) compromises planning and implementation of MDA campaigns and estimating their coverage. NTD drug inventory estimates are often based on the treatment registers as no dedicated and comprehensive system currently exists to track NTD supplies, and there is poor or no integration with national logistics management information systems (LMIS). Finally, though M&E data are usually of high quality, access and use of such data are inhibited by minimal system integrations and lack of feedback loops.

The articulation of challenges and limitations related to priority data sources and the subsequent expert convening led to the development of 12 use cases, from which eight use cases were prioritized by expert discussion and are shown in Table 1. Detailed descriptions of each use are provided elsewhere ( Grubin, 2021).

**Table 1.** Eight prioritized NTD data use cases.

Use Case	Objective	Key Actors	Critical Data Challenges	Causal Issues
**# 1. Improve** ** treatment ** **register data** ** quality**	Enable good decision making for stronger NTD country programs by improving the accuracy, comprehensiveness, timeliness, access, and use of treatment register data.	• Drug distributors	• Data accuracy • Data timeliness • Drug distributor workload	• Lack of appropriate data collection tools • High volumes of data • Insufficient training and incentives around data use • Insufficient or late stipend payments • Insufficient funds for operations
**# 2. Strengthen** ** supervision of ** **drug distributors**	Support drug distributors in meeting coverage targets through provision of consistent training and supervision	• Supervisors of drug distributors	• Data accuracy • Data timeliness • Drug distributor workload	• High volumes of data • Insufficient human resources at health facilities • Insufficient training for supervisors • Insufficient data tools or systems to support supervision
**# 3. Generate** ** accurate ** **community-level ** **population data** ** for MDAs**	Determine resources needed and coverage rates achieved for MDA campaigns by providing sufficiently accurate community-level denominator data	• Drug distributors • District NTD officers • National NTD program team members	• Data accuracy	• Lack of financial resources • Dependency on data sources of poor quality or insufficient granularity • Lack of appropriate tools
**# 4. Create** ** and manage** ** an accurate** ** inventory of drugs**	Manage drug supply for successful MDA campaigns effectively through improved accuracy, transparency, and access to NTD drug supply chain data (including reverse supply chain) at all levels of the health system	• NTD district and regional officer/ pharmacist • National NTD team members	• Data accuracy • Incomplete data • Lack of access to data	• NTD supply chain managed separately from national supply chain • Insufficient funds for operations to support reverse supply chain • Insufficient training for data management • Lack of appropriate data collection tools
**# 5. Meet district ** **coverage targets** ** during MDA** ** campaigns**	Assess progress towards coverage targets in real-time during MDAs, including determination of resources reallocation or mop-up activities, through improved access to sub-district NTD data for district NTD officers	• District NTD officer	• Cumbersome manual processes • Disparate/siloed databases and systems	• Insufficient data tools and systems for timely data sharing and analysis • Lack of human resources at the district level
**# 6. Feedback on** ** performance to** ** sub-district teams**	Create data and performance feedback loops to incentivize data use and improve implementation at sub- district level	• Drug distributors • First-line health workers • District NTD officer	• Lack of access to data and MDA results at sub-district levels • Data not used or valued by sub-district actors	• Data only flows one way, limiting access to data at sub-district levels • Insufficient tools or systems to support data sharing and feedback loops • Insufficient training and incentives around data use • Lack of human resources to support robust feedback loops
**# 7. Feedback** ** on performance** ** related to ** **monitoring ** **activities and data** ** quality provided** ** to sub-national** ** teams**	Create data and performance feedback loops to incentivise data use and improve implementation at sub- national level	• District NTD Officer • National NTD team	• Lack of access to data and MDA results at sub-national levels • Data not used or valued by sub- national actors	• Data only flows one way, limiting access to data at sub-national levels • Insufficient tools or systems to support data sharing and feedback loops • Insufficient training and incentives around data use • Lack of human resources to support robust feedback loops
**# 8. Improved ** **system ** **interoperability** ** for stronger** ** national-level ** **program use ** **of data for ** **evaluation and ** **decision making**	Effective evaluation and decision making for NTD programs through improved access to NTD and relevant HIS data, including analysis and visualization capabilities	• National NTD team	• Data stored in disparate databases and systems • Cumbersome manual processes • Systems not designed for long- term sustainability to support surveillance	• Insufficient data analysis and visualization tools to support decision making • Lack of system interoperability • Limited human resources • Limited financial resources

Two of the use cases address treatment registers (improving data collection and strengthening supervision of drug distributors), one looks to strengthen census data with the generation of accurate community-level population data, and one aims to create and manage an accurate drug ledger. The remaining four – meeting district coverage targets, ensuring both sub-district and sub-national teams receive feedback on performance, and improving national-level program use of data for evaluation and decision making – look to strengthen M&E survey data. The key actors involved in the data activities ranged from national NTD staff through district officers to drug distributors and first-line health workers. The main data challenges faced in each use case included lack of data access, low data accuracy and timelines, and siloed data systems. The causal factors underpinning all eight use cases are, in part, a result of the enabling environment and the maturity of the related components defined by the WHO building blocks of data systems ( WHO, 2010).

Table 2 presents the key solution profile for each use case. Improved data access and data quality were key features in many of the solution profiles. Strategies that reduced workload and enabled increased use and analysis of data were also important. Another theme was supported supervision and use of feedback loops at each level of the data system. Lastly, better integration of data systems within NTD programs and across the health system were also seen as important.

**Table 2.** Main solution profiles of the eight use cases.

Use Case	Solution profile
**# 1. Improve treatment** ** register data quality**	• *Data Quality*: improve the ability to measure data accuracy and comprehensiveness of treatment register data and track them over time with demonstrated improvement; • *Transmission Time*: improve timeliness of reporting treatment register data; • *Access*: increase access to dis-aggregated treatment register data for use by relevant stakeholders during and after an MDA campaign; and • *Reduced Workload*: streamline the work of data collection and reporting for drug distributors, contributing to an overall reduction in workload.
**# 2. Strengthen supervision ** **of drug distributors**	• *Adherence*: improve the effectiveness of training campaigns for drug distributors, thereby improving their adherence to guidelines; • *Supportive Supervision*: improve the provision of and access to supervision during an MDA and the ability to track related differences in drug distributor performance, ensuring targets are met and community members are not missed during MDA; and • *Data Quality*: improve the ability to measure data accuracy and comprehensiveness and to track them over time with demonstrated improvement.
**# 3. Generate accurate** ** community-level population** ** data for MDAs**	• *Flexibility*: have the ability to work for different actors, across various types of locations and working environments, each with potentially different levels of access to technology, power, connectivity, etc.; • *Access*: increase access to community-level census data for use by relevant stakeholders before, during, and after MDA campaigns; and • *Data Quality*: improve the ability to measure data accuracy and comprehensiveness and to track them over time with demonstrated improvement; • *Alignment*: all in keeping with national systems and other community health programs.
**# 4. Create and manage an** ** accurate inventory of drugs**	• *Transparency*: improve oversight for the drug inventory (stock levels, distribution, reallocation needs, etc.); • *Transmission Time*: improve timeliness of reporting drug inventory data; • *Access*: increase access to the drug ledger data and supply chain data tools for use by relevant stakeholders; • *Data Quality*: improve the ability to measure data accuracy and comprehensiveness of drug ledger data and track them over time with demonstrated improvement; and • *Integration:* ensure integration to existing LMIS and export into Joint Request for Select Medicines (JRSM) and Zithromax applications.
**# 5. Meet district coverage ** **targets during MDA** ** campaigns**	• *Transmission Time*: improve the timeliness of reporting aggregate treatment register data to the district level; • *Access*: increase access to the summary form treatment register data for use by relevant stakeholders during the MDA campaign; and • *Analysis*: have the ability to summarize and quantify data in a simple interface, allowing NTD District officers to view patterns and totals and use data for decision making.
**# 6. Feedback on ** **performance to sub-district** ** teams**	• *Data Aggregation & Visualization*: aggregate data from disperse data sets and enable users to quickly and effectively visualize data and create reports to share information with stakeholders at sub-district levels of the health system; • *Feedback Loops*: enhance bilateral communication between NTD district, health facility, and community-level actors, focused on providing feedback and information to project teams at each level; • *Access*: increase access to the data for use by relevant stakeholders; and • *Data Use*: include mechanisms and approaches to improving data use at sub-district levels of the health system.
**# 7. Feedback on** ** performance related to** ** monitoring activities and** ** data quality provided to sub-** **national teams**	• *Integration:* integrate with national systems and web-based access to promote secure accessibility by multiple users at different levels of the health system; • *Data Aggregation & Visualization*: aggregate data from disperse data sets and enable users to quickly and effectively visualize data and create reports to share information with stakeholders at other levels of the health system; • *Feedback Loops*: enhance bilateral communication between the national NTD team and NTD regional and district teams, focused on providing feedback and information to project teams at each level; • *Access*: increase access to the data for use by relevant stakeholders; and • *Data Use*: include mechanisms and approaches to improving data use at sub-national levels of the health system.
**# 8. Improved system** ** interoperability for stronger** ** national-level program use** ** of data for evaluation and ** **decision making**	• *Integration*: import or access data from disparate data sets that may be stored in different paper or electronic systems (e.g., routine MDA data, drug inventory data, morbidity data, impact evaluation surveys, non-NTD data sets held in national systems that are relevant to NTD program management) to facilitate centralized access to these data and improved data use; • *Data Analysis and Visualization*: enable users to quickly and effectively conduct analysis and data visualization to enhance data use and support decision making; and • *Data Quality*: improve the ability to measure data accuracy and comprehensiveness and to track them over time with demonstrated improvement.

## Discussion

These eight use cases illustrate the level of specificity required to understand the needs of NTD program actors making decisions as well as the systematic bottlenecks that prevent the effective use of data. Each use case highlights the parallel existence of system-specific and enabling environment challenges that prevent the ready access to and effective use of data. Effective system design requires focusing on actor needs from the start, including clear articulation of what data will be collected, by whom, how, and for what purpose, in turn ensuring data are usable and being used for decision making. The successful completion of these use cases would result in improved data quality, access, and use for NTD actors across the different levels of the health system.

The development of any tool or system will need to take local context into account ( Novillo-Ortiz *et al*., 2018). For some programs, the introduction of digital solutions where appropriate could bring the most benefit, while for others improving operational processes or streamlining paper-based reporting may bring the most value. Consideration for how the use cases fit together is also critical, as introducing tools to support analysis and visualization are of little value if the data inputs are unreliable or incomplete. The recent COVID-19 pandemic has introduced an additional complexity for introducing data solutions, but there also may be opportunities to leverage some of the data solutions introduced for COVID-19 responses. 

**Figure 2.**
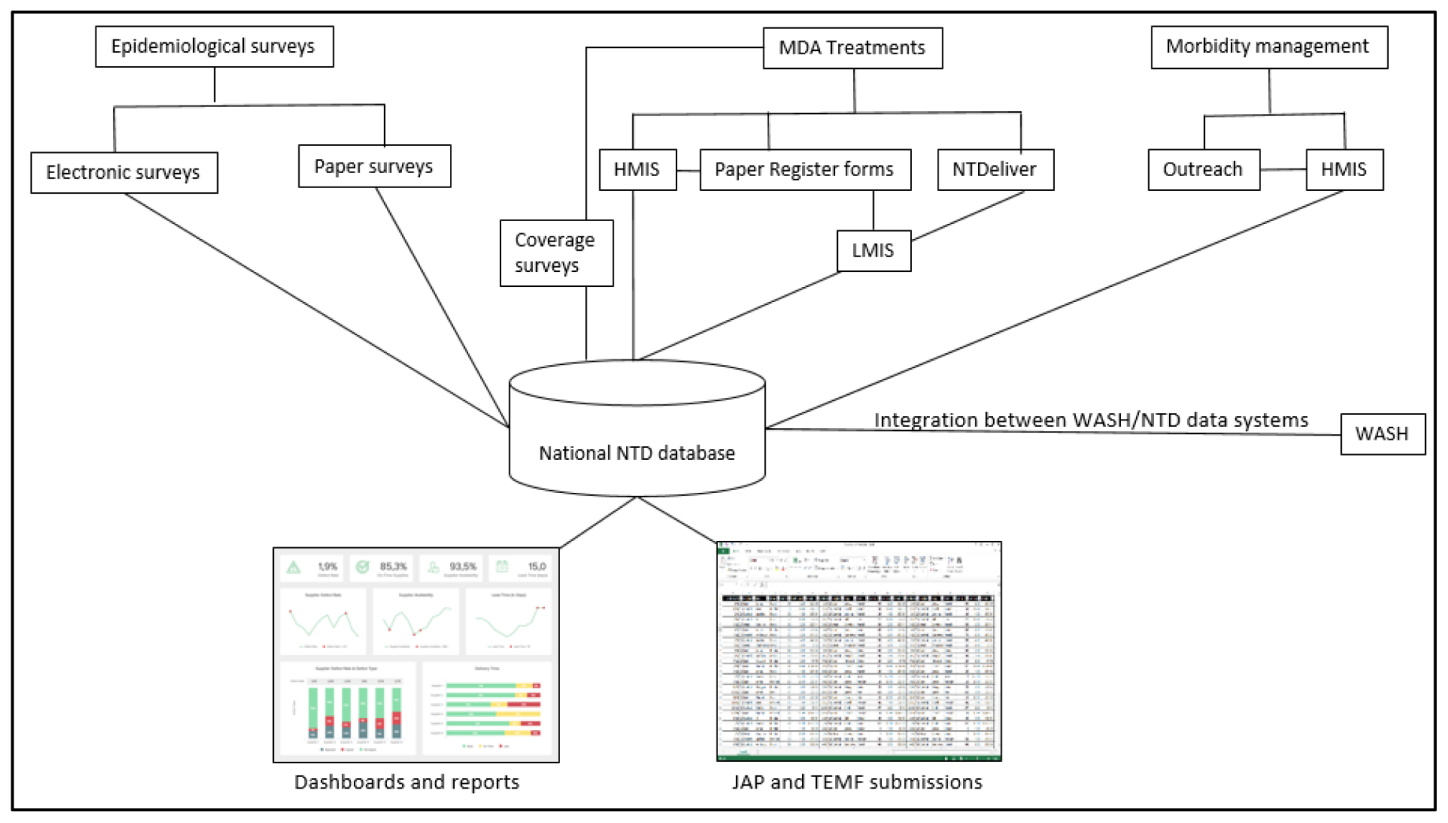
Illustrative example of an integrated NTD system.

Additionally, the use cases emphasize the value of data systems that are integrated across the different NTDs and with national HIS. Any integrated systems should capture complete, timely, accurate, and disaggregated (at least by location, age, and gender) data, provide tools for data collection and analysis, and enable standardized, online reporting through harmonized data platforms such as the ESPEN Data Portal ( ESPEN, 2020). An integrated system would avoid disease-specific databases and separate reporting by different stakeholders and donors. An example of an integrated system is presented in Figure 2. Here, administrative units, population, treatments, and morbidity are tracked by the national HIS; drug supplies and inventory are tracked by the LMIS; and epidemiological surveys, treatment coverage surveys, and water, sanitation, and hygiene data are aggregated into a national NTD database. Such a system would allow for data use by different users at multiple levels, thereby minimizing the risk of data divergence across different NTD data systems.

New tools and systems alone will not be sufficient to ensure that data are used effectively by NTD actors in their decision-making and activities. There is a need to strengthen the capacity to analyze and use data at all levels of the NTD program. Part of the solution is to create a culture of data use in the NTD sector. Innovations implemented to date have demonstrated the usefulness of data review sessions to improve the quality of data ( de Souza *et al.,* 2016). Each of the use cases presented here provides opportunities to empower NTD programs from district to national levels to use data to inform their decision-making.

The systematic strengthening of data use for decision-making in NTD programs is key for reaching the 2030 Roadmap goals. Integrated together, the presented use cases, when translated into actions using appropriate and innovative solutions, can help to ensure that accurate and timely data are present at every step of a program and empower countries to use these data to make program decisions.

## Data availability

### Underlying data

Harvard Dataverse: Improving data use for decision making by neglected tropical disease programs. Detailed description of NTD use cases, https://doi.org/10.7910/DVN/SPZGFU.

This project contains the following underlying data:

- Data file 1. (Detailed description of NTD data use cases. (2021-10-22)

Data are available under the terms of the Creative Commons Zero "No rights reserved" data waiver (CC0 1.0 Public domain dedication).
