# Supplementary material for: Improving data use for decision making by neglected tropical disease program teams: eight use cases
Source: Gates Open Res. 2021 Nov 9;5:153. [Version 1] doi: 10.12688/gatesopenres.13407.1 (PMC8666716; doi:10.12688/gatesopenres.13407.1)
Supplement: Peer review history

# Peer review: Reviewer response for version 1

**DOI:** 10.21956/gatesopenres.14661.r31398

**Published:** 2021-12-08

**Authors:** Collins Okoyo

**Affiliations:** 1 Eastern and Southern Africa Centre of International Parasite Control, Kenya Medical Research Institute (KEMRI), Nairobi, Kenya

The paper “Improving data use for decision making by neglected tropical disease program teams: eight use cases” is well presented in general. The authors have discussed the results in a balanced way. Below are some general comments for your consideration. Additional comments are highlighted within the annotated manuscript which can be found here:

 Introduction:

- State why the eight use cases were only applied to the 5 NTDs (LF, Oncho, SCH, STH and Trachoma) but not other NTDs.
- Justify why the focus (data gathering) was made only for three countries (Burkina Faso, Ethiopia and Tanzania) and describe their current disease burden with regards to the 5 NTDs.

Methods:

- During phase 2 (assessment of country level use of NTD data), how were the participants of interest (program managers, data managers, pharmacists, health workers, drug distributors and teachers) sampled for inclusion in the study?
- Please indicate whether the study received ethical approval or if this requirement was waivered.
- Please indicate how the qualitative data collected from the participants was managed and analyzed.

Is the work clearly and accurately presented and does it cite the current literature?

Yes

If applicable, is the statistical analysis and its interpretation appropriate?

Partly

Are all the source data underlying the results available to ensure full reproducibility?

Yes

Is the study design appropriate and is the work technically sound?

Yes

Are the conclusions drawn adequately supported by the results?

Yes

Are sufficient details of methods and analysis provided to allow replication by others?

Partly

Reviewer Expertise:

Statistics; Data management; Infectious disease epidemiology; Neglected tropical diseases; Public health; Mathematical modeling

I confirm that I have read this submission and believe that I have an appropriate level of expertise to confirm that it is of an acceptable scientific standard.

# Peer review: Reviewer response for version 1

**DOI:** 10.21956/gatesopenres.14661.r31399

**Published:** 2021-11-24

**Authors:** Katherine M. Gass

**Affiliations:** 1 Neglected Tropical Disease Support Center, Task Force for Global Health, Decatur, GA, USA

This article provides a comprehensive summary of the ways in which NTD programs collect and use data to make decisions. The authors used a series of collaborative processes to engage national NTD programs, implementing partners, and global stakeholders to understand the generation and use of NTD data. This information was used to map the flow of MDA campaign data and led to the identification of broad themes for how NTD programs use data and the data sources that are most pivotal to decision making. A prioritization process was used to determine the top 8 use cases for NTD data, the critical challenge preventing the full realization of these use cases and the causal issues behind the challenges. Finally, a solution profile was generated for each use case, elucidating several cross-cutting themes such as data quality, timeliness, transparency, access, integration, and visualization and analysis. 

 This article was well-written, succinct in its presentation and delivers timely information. It is clear that data related challenges are, to varying degrees, holding NTD programs back from making more appropriate, accurate, efficient or timely decisions. I appreciate that this review of data processes covered all aspects of NTD program data (from registers to drug ledgers to M&E data) and involved input from in-country stakeholders at the district and community level. The Figure 1 in this article, which depicts MDA campaign data flow, helps to clarify and illustrate the complexity of NTD data flow and should serve as a useful primer for anyone entering NTD program work. 

 My wish for this article is that it can lead to a call to action around concrete steps that NTD programs and their stakeholders can take to relieve these data bottlenecks. The solutions outlined for each use case are helpful in calling out the broad solutions required but lack the specificity needed to implement them locally.

Is the work clearly and accurately presented and does it cite the current literature?

Yes

If applicable, is the statistical analysis and its interpretation appropriate?

Not applicable

Are all the source data underlying the results available to ensure full reproducibility?

Partly

Is the study design appropriate and is the work technically sound?

Yes

Are the conclusions drawn adequately supported by the results?

Yes

Are sufficient details of methods and analysis provided to allow replication by others?

Partly

Reviewer Expertise:

NTD epidemiologist, survey design, monitoring and evaluation

I confirm that I have read this submission and believe that I have an appropriate level of expertise to confirm that it is of an acceptable scientific standard.

# Peer review: Reviewer response for version 1

**DOI:** 10.21956/gatesopenres.14661.r31396

**Published:** 2021-11-15

**Authors:** Dziedzom K. de Souza

**Affiliations:** 1 Department of Parasitology, Noguchi Memorial Institute for Medical Research, University of Ghana, Accra, Ghana

This paper presents the important description of eight use cases of NTD data to improve the activities and decision making of different stakeholders for the five main PC NTDs. It is very well written. A couple of additional inputs/clarifications will improve it quality. 

 Introduction, last paragraph: - Perhaps, it should be indicated that these use cases target the PC NTDs, as the data use cases for CM NTDs might vary.

 The list of all the 20 potential use cases identified at the expert strategic convening in 2018 should be provided as a table. They many not be relevant for this paper, but identifying these use cases may enable other actors to act accordingly depending on the local needs.

 Were the eight selected use cases a combination of the previous 20 identified? What informed the selection of the eight use cases over others?

 Figure 1: I see a potential issue at the health facility level. If the coverage target is not met, drug distribution is usually not done again. The figure makes it seem that is usually the case, but NTD programmes have limited periods within which to conduct the MDA. The tools available (e.g. SCT) to ensure adequate coverage are usually implemented during the MDA.

 Figure 1: Perhaps the different survey options use for the QC/QC procedures (SCT, CES, DQA-S, etc.) and where along the health system they can be implemented should be indicated in the Figure (or a separate figure).

 Figure 1: QA/QC is also done at the regional level.

 Figure 1: It might be useful to include a 6th level (INTERNATIONAL) to reflect the data use by WHO and NTD funding partners. This level also determines the drug requests and funding needs.

 Figure 1: Ideally, QA/QC should also be done at the community level. While the overall data at the district level may balance out and reflect an effective coverage at the district level, individual community level coverages may not be optimal. Further, data inaccuracies at the community level will be aggregated throughout the entire system to the national level. This should also be considered as a solution in Table 2.

 A critical use case not identified is the identification of MDA non-compliants (and systematic non-compliants or "Never Treated"). Registers do provide the opportunity to identify such individuals. As one of the critical challenges to MDA, the use of data to address non-compliance levels in a population should be addressed.

 Table 2: The time and funding for MDA are two important components that need to be considered/discussed in the challenges and the solutions. It is known that the time to conduct MDA (usually 2 weeks) is a limiting factor for an effective MDA campaign. Funding by partners and reporting timelines also force NTD programmes to restrict activities within a certain time frame. As such the solutions should include extending the timing of activities and not limiting it to the demands of the international donor community.

 Discussions: The critical roles of external stakeholders (WHO, funding partners, etc.), and their data needs and uses, must be discussed as this influences activities in the countries.

Is the work clearly and accurately presented and does it cite the current literature?

Yes

If applicable, is the statistical analysis and its interpretation appropriate?

Not applicable

Are all the source data underlying the results available to ensure full reproducibility?

No source data required

Is the study design appropriate and is the work technically sound?

Yes

Are the conclusions drawn adequately supported by the results?

Yes

Are sufficient details of methods and analysis provided to allow replication by others?

Yes

Reviewer Expertise:

Lymphatic filariasis, NTDs

I confirm that I have read this submission and believe that I have an appropriate level of expertise to confirm that it is of an acceptable scientific standard, however I have significant reservations, as outlined above.
